# An Efficient Visual Screen for CRISPR/Cas9 Activity in *Arabidopsis thaliana*

**DOI:** 10.3389/fpls.2017.00039

**Published:** 2017-01-24

**Authors:** Florian Hahn, Otho Mantegazza, André Greiner, Peter Hegemann, Marion Eisenhut, Andreas P. M. Weber

**Affiliations:** ^1^Institute of Plant Biochemistry, Cluster of Excellence on Plant Science, Center for Synthetic Life Sciences, Heinrich Heine UniversityDüsseldorf, Germany; ^2^Institute of Biology, Experimental Biophysics, Humboldt-Universität zu BerlinBerlin, Germany

**Keywords:** CRISPR/Cas9, trichome, Glabrous1, Glufosinate, BASTA, gene editing, marker

## Abstract

The CRISPR/Cas9 system enables precision editing of the genome of the model plant *Arabidopsis thaliana* and likely of any other organism. Tools and methods for further developing and optimizing this widespread and versatile system in *Arabidopsis* would hence be welcomed. Here, we designed a generic vector system that can be used to clone any sgRNA sequence in a plant T-DNA vector containing an ubiquitously expressed *Cas9* gene. With this vector, we explored two alternative marker systems for tracking Cas9-mediated gene-editing *in vivo*: *BIALAPHOS RESISTANCE* (*BAR*) and *GLABROUS1* (*GL1*). *BAR* confers resistance to glufosinate and is widely used as a positive selection marker; *GL1* is required for the formation of trichomes. Reversion of a frameshift null *BAR* allele to a functional one by Cas9-mediated gene editing yielded a higher than expected number of plants that are resistant to glufosinate. Surprisingly, many of those plants did not display reversion of the *BAR* gene through the germline. We hypothesize that few *BAR* revertant cells in a highly chimeric plant likely provide system-wide resistance to glufosinate and thus we suggest that BAR is not suitable as marker for tracking Cas9-mediated gene-editing. Targeting the *GL1* gene for disruption with Cas9 provided clearly visible phenotypes of partially and completely glabrous plants. 50% of the analyzed T1 plants produced descendants with a chimeric phenotype and we could recover fully homozygous plants in the T3 generation with high efficiency. We propose that targeting of *GL1* is suitable for assessing and optimizing Cas9-mediated gene-editing in *Arabidopsis*.

## Introduction

A growing world population facing increasingly scarce arable land would benefit from applying synthetic biology approaches to generate rationally designed plants with low effort and within short time frames (Baltes and Voytas, 2015). Recently, it became possible to modify plant genomes by introduction of double-strand breaks (DSBs) at a locus of interest, using site-specific custom nucleases. DSBs are lethal for the cell and must be repaired by either homologous recombination (HR) or non-homologous end joining (NHEJ), which are carried out by plant internal DNA break repair mechanisms (Puchta and Fauser, 2014). Both mechanisms can be exploited for targeted genome editing. While HR enables the targeted integration of sequences of choice into the DSB mediated by homologous regions between a DNA donor template and the target region, the error-prone NHEJ can be exploited to destroy open reading frames of genes and thus to disrupt gene function (Salomon and Puchta, 1998). The development of the CRISPR/Cas9 (hereafter Cas9) system, which relies on the bacterial Cas9 nuclease from *Streptococcus pyogenes* and a single guide RNA (sgRNA) molecule that directs the nuclease to its specific target (Jinek et al., 2012) has greatly reduced the effort to create DSBs at specific loci in the genome of living cells (Doudna and Charpentier, 2014). The Cas9 system allows the creation of DSBs in virtually any gene and even in several genes simultaneously. It has already been successfully applied in many model and crop plants species (Bortesi and Fischer, 2015). Cas9 has quickly established itself as a powerful and versatile technology and strong efforts are made on developing and optimizing Cas9 related tools and methods. However, markers as indicators for the successful mutagenesis are still scarce.

A commonly used marker for positive selection of transgenic plants is resistance to the broad-spectrum herbicide glufosinate also known as phosphinothricin or BASTA^®^ (Deblock et al., 1987). Glufosinate is a glutamate analog that inhibits glutamine synthetase *in planta* and thus hinders the conversion of glutamate and ammonia to glutamine (Bayer et al., 1972; Lea et al., 1984). The accumulation of ammonia in the plant inhibits photosynthetic reactions and uncouples photophosphorylation, finally resulting in cell death (Tachibana et al., 1986; Sauer et al., 1987; Wild et al., 1987). Resistance to glufosinate is conferred by the bialaphos resistance gene (*BAR*) from *Streptomyces hygroscopicus*, which encodes the enzyme phosphinotricin acetyl transferase (PAT). PAT acetylates the amino group of glufosinate and thereby inactivates it (Thompson et al., 1987; Mullner et al., 1993). Since the *BAR* gene has been highly successfully applied as positive selection marker for screening of T-DNA insertion lines, a *BAR*/glufosinate based screening could be implemented also for assessing Cas9 based gene-editing in plants.

The genes that control trichome development could be used as alternative non-invasive markers for visually tracking mutations in the model plant *Arabidopsis thaliana*. Trichomes are specialized epidermal cells, which are found on most aerial parts of the plant. They function in protection against UV light, herbivores and pathogen attack, extreme temperatures, and water loss (reviewed in Yang and Ye, 2013; Pattanaik et al., 2014). Importantly, they are not essential for plant growth and development under laboratory conditions (Oppenheimer et al., 1991). The development and distribution of trichomes on the leaf surface are highly regulated processes. In *Arabidopsis*, trichome initiation starts very early during leaf development with an increase in cell and nuclear size by at least four rounds of endoreplication. The trichome cell then elongates and branches. While the first trichomes form at the distal region of the leaf primordium, subsequent trichomes develop at the more basal region of the leaf primordium and in between the mature trichomes on the distal parts (Larkin et al., 1994, 1996; Marks, 1994). Trichome development is strongly reduced by knocking out the gene for the R2R3-MYB transcriptional master regulator GLABROUS1 (GL1) (Koornneef et al., 1982). Like other plant MYB proteins, GL1 contains two MYB DNA-binding domain repeats at the N-terminus and an acidic C-terminus (Larkin et al., 1993). GL1 interacts with the WD40-repeat factor TTG1 and the bHLH factor GL3/EGL3 to form a MYB/bHLH/WD-repeat complex. This complex activates the expression of numerous downstream activators, such as TTG2 and GL2, which allow epidermal cells to differentiate into trichomes (Yang and Ye, 2013; Pattanaik et al., 2014). Homozygous *gl1* mutants have been described to be completely or partly glabrous or showing at least a reduced number of trichomes (Koornneef et al., 1982), depending on the strength of the mutational effect. Mutations in the MYB DNA-binding domains seem to produce the most drastic phenotypes (Hauser et al., 2001).

In this report, we tested and compared the positive selection marker BAR, which confers resistance to glufosinate, and the non-pleiotropic visual marker GL1 as markers for Cas9 activity in *Arabidopsis*. Therefore, we first created a vector system that can be used to clone any sgRNA into a plant T-DNA vector that contains the *Cas9* gene under the control of the *UBIQUITIN10* promoter. Using this vector system, we were able to repair an inactive *bar* allele that carries a premature stop codon in *Arabidopsis* using Cas9 and to positively select plants by glufosinate treatment. However, low incidence of the repaired allele in the leaves of the plants resistant to BASTA suggests that few cells producing a functional BAR protein are sufficient to rescue the whole plant by conferring some degree of system-wide resistance. This low stringency of the selection system did not allow detection of homozygous mutants in the following generations. We were also able to knock out the *GL1* gene using the Cas9 technique. With a *GL1* specific sgRNA we generated several independent glabrous *Arabidopsis* lines. Genotyping showed that small InDels at the *GL1* target site caused shifts in its reading frame and premature stop codons resulting in a disruption of the GL1 protein function. With the *gl1* phenotype, we provide an efficient visual screen that can be used for testing mutagenesis efficiency under various experimental conditions in further studies.

## Materials and Methods

### Plant Growth Conditions

*Arabidopsis* Col-0 seeds were surface-sterilized and stratified for 3 days at 4°C. Seeds were germinated on 0.8% (w/v) agar-solidified half-strength MS medium (Duchefa, http://www.duchefa.com) supplemented with 1% (w/v) sucrose under long day conditions (16-h-light/8-h-dark cycle, 22/18°C) in growth chambers with a light intensity of 100 μmol photons m^-2^ s^-1^. 14-day-old seedlings were transferred to soil and grown under the same conditions.

### Isolation of a Homozygous *gl1* T-DNA Insertion Line

T-DNA insertion lines are a common way to analyze the impact of gene knockouts in *Arabidopsis*. The T-DNA insertion line *gl1* (SAIL_1149_D03) (Sessions et al., 2002) that contains a T-DNA integration in between the first and second exon of the *GL1* gene was obtained from the Arabidopsis Biological Resource Centre. Homozygous T-DNA insertion plants were identified using the gene specific primer pair FH214 and FH215 (Supplementary Table S1) as well as the T-DNA specific primer pair FH215 and P49 (Supplementary Table S1). Homozygosity was additionally confirmed by visual control of trichome loss. Homozygous *gl1* plants were further propagated.

### Generation of a Binary T-DNA Vector with the Null *bar-1* Allele

To construct a binary T-DNA vector with a null *bar-1* allele, we first amplified the *BAR* gene from the vector pUB-Dest (Grefen et al., 2010) with the primer pair FH5/FH6 and cloned the PCR product into pDONR207 via BP reaction (Invitrogen) resulting in the vector pFH24. We then created an internal stop codon in the *BAR* gene via overlapping PCR. Therefore, we first amplified the 5′-end of the *BAR* gene with the primer pair FH25/FH26 and the 3′-end with primer pair FH27/FH28 from pFH24 and then used both PCR products as PCR template with the primer pair FH25/FH28. The PCR product was subcloned into pJET1.2 (ThermoFisher Scientific) resulting in the vector pFH10. Finally, we digested the plant binary T-DNA vector pUT-Kan (Fendrych et al., 2014) with PstI and BamHI, PCR-amplified the *BAR* gene with the internal stop codon from pFH10 with the primer pair FH39/FH40 and assembled the final vector pFH17 via Gibson cloning (NewEngland Biolabs). All primer sequences are given in Supplementary Table S1. The sequence of pFH17 can be found on GenBank (accession number KY080690).

### Cas9 and sgRNA Plasmid Construction

**FIGURE 1:**
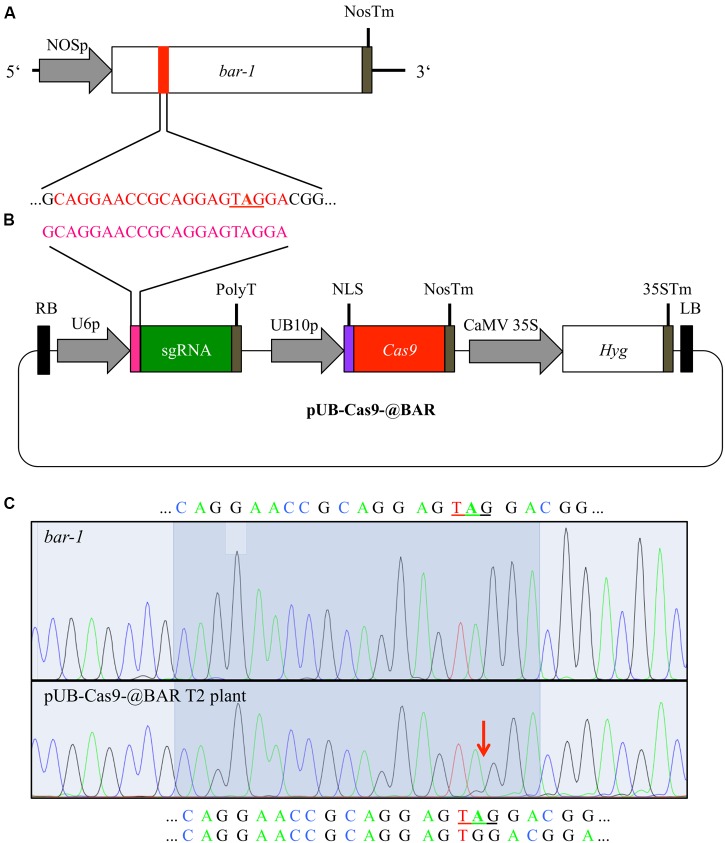
**Cas9-mediated repair of a disrupted *bar* gene**. We generated an *Arabidopsis* line with a null allele of the *BAR* gene (*bar-1*) that contains an extra adenine (**A** in bold) leading to a premature STOP codon **(A)**. In order to induce mutations, *Arabidopsis* plants homozygous for the *bar-1* insertion were transformed with the single T-DNA construct pUB-Cas9-@BAR **(B)** which contains the sgRNA (green) with the 20 bp target motive (pink) and an additional guanine at the beginning (necessary for transcription) under the control of the *U6-26* promoter (U6p), the *Cas9* (red) controlled by the *UBIQUITIN10* promoter (UB10p) and the selection marker hygromycin (*Hyg*). Gene sizes are not to scale. T2 transformants were then treated with glufosinate and resistant plants were analyzed for rescuing mutations in the *bar* gene in leaf genomic DNA. The direct comparison of sequencing chromatograms **(C)** of a glufosinate resistant plant (bottom) and one of a control plant containing only the *bar-1* allele (top) shows small additional peaks appearing at the expected site of Cas9 induced mutagenesis (red arrow) at 3 bp in front of the PAM site in the glufosinate resistant plant. The 20 bp target site is highlighted and the validated sequence is given for the null allele plant (top) and the resistant plant (bottom; first line: main peaks, second line: minor peaks). The premature stop codon is underlined. NOSp, nopaline synthase promoter; NosTm, nopaline synthase terminator; RB, right T-DNA border; PolyT, poly thymidine transcription terminator; NLS, nuclear localization signal; CaMV 35S, *Cauliflower Mosaic Virus* 35S promoter; 35STm, 35S terminator; LB, left T-DNA border.

We aimed at generating a single T-DNA vector for expression of the Cas9 protein and the sgRNA cassette. Therefore, a *Chlamydomonas reinhardtii* codon-optimized *Sp*Cas9 was amplified with the primers NH117/NH119 (Supplementary Table S1) from the vector CasYFP and cloned under the control of a *UBIQUTIN10* promoter (*UB10p*) from *Arabidopsis* into the vector pKB65 (Bernhardt, 2012) via Gateway^®^ Cloning (Invitrogen) resulting in the vector pUB-Cas9. For sgRNA expression, the *Arabidopsis U6-26* promoter (*U6-26p*) was PCR-amplified with an additional NheI restriction site from *Arabidopsis* genomic DNA using the primers FH14 and FH15 (Supplementary Table S1). The PCR fragment was subcloned into the pGEM T-easy^TM^ vector (Promega) resulting in the vector pFH13. The sgRNA scaffold was amplified from the vector pKS Chlamy dual with the primers FH16 and FH18 (Supplementary Table S1) and subcloned into pJET1.2 (ThermoFisher Scientific) resulting in the vector pFH4. To create a sgRNA expression cassette with the sgRNA scaffold and the *U6-26p*, the *U6-26p* was PCR-amplified from pFH13 with FH21 and FH22 (Supplementary Table S1) and the sgRNA scaffold was PCR-amplified from pFH4 with FH23 and FH24 (Supplementary Table S1). The two PCR products were combined with NheI/SpeI-digested pFH13 to the construct pFH6 via Gibson assembly^®^ (NEB). To introduce the 20 bp target sequence designed against the *GL1* gene, pFH6 was digested with BbsI (NEB) and the vector backbone was ligated with two annealed primers (FH35/FH36, Supplementary Table S1) containing the target sequence resulting in the vector pFH26. The final T-DNA transformation vector pUB-Cas9-@GL1 (with the @ representing the targeted gene) was constructed by Gibson Assembly^©^ (New England Biolabs) of KpnI and HindIII-digested pUB-Cas9 and the sgRNA cassette that was amplified from pFH26 with FH41 and FH42 (Supplementary Table S1). Similarly, a target sequence designed against the disrupted *bar* gene was prepared. Since, the U6 RNA polymerase III promoter takes guanine as transcription start nucleotide, we could not use the usual 20 bp protospacer sequence. We therefore added an extra G at the 5′-end of the sgRNA (5′-GCAGGAACCGCAGGAGTAGGA-3′, **Figure 1B**) that should not interfere with Cas9 activity (Belhaj et al., 2013; Ran et al., 2013). The target sequence was created by annealing the two primers FH145/FH146 (Supplementary Table S1) and ligation into BbsI-digested pFH6 resulting in the vector pFH25. Gibson Assembly^©^ of KpnI and HindIII-digested pUB-Cas9 and the sgRNA cassette that was amplified from pFH25 with the primers FH41 and FH42 (Supplementary Table S1) resulted in the final T-DNA transformation vector pUB-Cas9-@BAR. The sequence of the vectors CasYFP (accession number KY080688), pKS Chlamy dual (accession number KY080687), pFH6 (accession number KY080689), pUB-Cas9 (accession number KY080691), pUB-Cas9-@GL1 (accession number KY080693) and pUB-Cas9-@BAR (accession number KY080692) can be found on GenBank.

### Plant Transformation

The T-DNA vectors pFH17, pUB-Cas9-@BAR and pUB-Cas9-@GL1 were used to transform *Agrobacterium tumefaciens*, strain GV3101::pMP90. *Arabidopsis* Columbia-0 (Col-0) wild type plants (WT) were then transformed by floral dipping as described in Clough and Bent (1998) with pFH17 and pUB-Cas9-@GL1, respectively. Primary transformants were selected using kanamycin (pFH17) or hygromycin (pUB-Cas9-@GL1). Additionally, genomic DNA was isolated from leaves of surviving plants via isopropanol precipitation (Weigel and Glazebrook, 2009) and the presence of T-DNA insertion was verified via PCR using plant genomic DNA as template and the primer pairs P16/P98 (Supplementary Table S1; pFH17) or FH158/FH159 (Supplementary Table S1; pUB-Cas9-@GL1).

Similarly, the vector pUB-Cas9-@BAR was transformed by floral dipping in *Arabidopsis* Col-0 plants homozygous for the insertion of the T-DNA vector pFH17 containing the *bar-1* null allele of the *BAR* gene. Primary transformants were selected on hygromycin and kanamycin. T-DNA insertion was verified via PCR with the primer pair FH61/FH201 (Supplementary Table S1) using plant genomic DNA as template.

### Molecular Determination of Cas9-Mediated Gene Editing

For detection of mutations in the pUB-Cas9-@BAR lines, plants were grown on soil under long day conditions. After 3 weeks, the plants were sprayed for 2 weeks with glufosinate ammonium (120 mg/L) every 3 days to isolate mutants that repaired the inactivated reading frame of the *BAR* gene via Cas9-mediated gene editing. Genomic DNA was isolated from leaves of surviving plants. The *BAR* gene was PCR-amplified with the primer pair FH154/FH155 (Supplementary Table S1) using the proofreading Phusion^©^ polymerase (NewEngland Biolabs) and the obtained PCR amplicon was sent for Sanger sequencing (Macrogen). Sequencing histograms were compared using 4Peaks software.

For mutation detection in the pUB-Cas9-@GL1 lines, plant genomic DNA was isolated and the targeted region in the *GL1* gene was amplified with the primers FH189/FH190 (Supplementary Table S1) using the proofreading Phusion^©^ polymerase (NewEngland Biolabs). The PCR product was then either digested with DdeI (NewEngland Biolabs) for detection of restriction digest length polymorphisms or Sanger sequenced (Macrogen). We used the CasOFF-Finder program (Bae et al., 2014) to detect putative off-target sites. For detection of mutations in the off-target *AT1G08810*, the putatively mutated region was amplified with primers FH258/FH259 (Supplementary Table S1) and then processed as described before.

### Detection of pUB-Cas9-@GL1 T-DNA in Glabrous Plants

Genomic DNA was isolated from leaves. Presence of the T-DNA was verified with primers amplifying specifically the *Cas9* gene (FH61/FH201, Supplementary Table S1) or the sgRNA gene (FH14/FH36, Supplementary Table S1). The T-DNA vector pUB-Cas9-@GL1 was used as positive control. High quality of the genomic DNA was verified via PCR on the housekeeping gene *Actin7* (*At5g09810*) using the primers P67/P68 (Supplementary Table S1).

## Results

We aimed at testing and evaluating markers for easy detection of Cas9 induced mutagenesis. Therefore, we explored the two alternative markers, resistance to glufosinate and development of glabrous leaves.

### Plants Carrying a Mutated Allele of the *BAR* Gene Are Not Resistant to Glufosinate

Glufosinate resistance is a well-established marker for positive selection of plants (Miki and McHugh, 2004) and allows screening of large plant populations by simple spraying of glufosinate. For a positive selection screening system, we first produced a mutant allele of the *BAR* gene (from here on called *bar-1*) and stably transformed *Arabidopsis* Col-0 plants with it. The *bar-1* allele contains an adenine insertion, which leads to a premature STOP codon (**Figure 1A**). We verified that this allele is indeed non-functional and does not confer resistance to glufosinate by growing homozygous transformants on soil for 3 weeks and treating them several times with glufosinate spraying. Out of 77 homozygous plants, none survived the treatment (Supplementary Figure S1).

### Design of a Single Generic Cas9 Vector for *Arabidopsis*

We wanted to design a vector system that can be used for targeted knockout of any gene in *Arabidopsis*. Therefore, we designed first the binary vector pUB-Cas9 encoding a Cas9 controlled by the *UBIQUITIN10* (*UB10)* promoter and containing insertion sites for the integration of the sgRNA cassette as well as a hygromycin resistance cassette for selection of transformants. Second, we designed a subcloning vector containing the *U6-26p*, followed by two BbsI-sites for protospacer integration and the sgRNA scaffold. After integration of the target sequence, any complete sgRNA cassette can basically be amplified via PCR using the same primer pair and be introduced in the binary vector pUB-Cas9 via Gibson cloning.

### Design of CRISPR/Cas9 System for Repairing the Mutant *BAR* Gene

In order to repair the mutated *bar-1* allele previously generated in *Arabidopsis*, we used the binary T-DNA vector pUB-Cas9-@BAR (**Figure 1B**). We designed our target sequence so that the Cas9 nuclease was expected to cut next to the adenine insertion in the *bar-1* gene, which causes the premature STOP codon, 3 bp upstream of the PAM motive. As small InDels were expected to occur there by incorrect NHEJ, we expected almost certainly a reading frame restoration of the *BAR* gene.

### T2 Plants Show High Survival Rate after Glufosinate Treatment

We transformed *Arabidopsis* plants homozygous for the *bar-1* allele with the pUB-Cas9-@BAR construct. We selected two positive transformants in the T1 generation. From both independent lines, we grew 77 T2 plants on soil and treated them after 3 weeks with repetitive glufosinate spraying. In both lines, the majority of plants (99 and 96%, respectively) survived the glufosinate treatment displaying different degrees of resistance to glufosinate (Supplementary Figure S1).

### Mutation Analysis Shows Only Minor Mutational Events

We amplified the *BAR* gene from leaves of 12 T2 glufosinate resistant plants from each line and sequenced the PCR product. As a control, we also amplified the *BAR* gene from a homozygous *bar-1* knockout plant that was not transformed with the pUB-Cas9-@BAR construct. All sequencing chromatograms displayed the sequence of the defective *bar-1* allele in the main peaks. However, starting at the Cas9 target sequence, minor chromatogram peaks were often visible, confirming that indeed Cas9 was actively targeting and inducing mutations in the *bar-1* reading frame (**Figure 1C**; Supplementary Figure S2A). In two of the 24 analyzed plants, we could conclude from the sequencing chromatograms that the inserted adenine was deleted in a part of the cells and the reading frame of the *BAR* gene was restored (**Figure 1C**). In the other plants, either additional small InDel mutations occurred that did not restore the reading frame or no change compared to the control DNA was visible (Supplementary Figure S2). This result indicated that even though most cells were not able to produce a functional PAT protein, resistance to glufosinate was still achieved.

### Generation of Homozygous Mutants Was Not Successful

We analyzed 8 T3 plants from the progeny of two T2 pUB-Cas9-@BAR plants that were resistant to glufosinate. We searched for mutations in the *bar* gene as described above. As with the T2 generation, we obtained sequencing chromatograms that mainly displayed the sequence of the *bar-1* allele with additional peaks starting at 3 bp in front of the PAM motive (Supplementary Figure S2A). We could not obtain non-chimeric plants with a fully restored *bar* gene.

### The *gl1* T-DNA Knockout Line Displays the Glabrous Phenotype

As the positive selection system using glufosinate did not yield completely mutated plants, we tested alternatively the non-invasive marker gene *GL1*. The *GL1* gene was chosen as target of Cas9 induced mutagenesis since *gl1* knockout plants are not able to form leaf and stem trichomes (Koornneef et al., 1982) and provide therefore a suitable visual marker to track mutation progress *in planta*. To establish a visual reference of the expected glabrous phenotype, an *Arabidopsis gl1* T-DNA insertion line was isolated (Supplementary Figure S3) and grown in parallel with *Arabidopsis* WT plants, ecotype Col-O. As expected, homozygous insertion lines did not produce trichomes on leaves and stems (Supplementary Figure S3). Additional differences in growth or development could not be detected as expected from previous reports (Oppenheimer et al., 1991).

### Design of Cas9 System for Editing *GL1*

**FIGURE 2:**
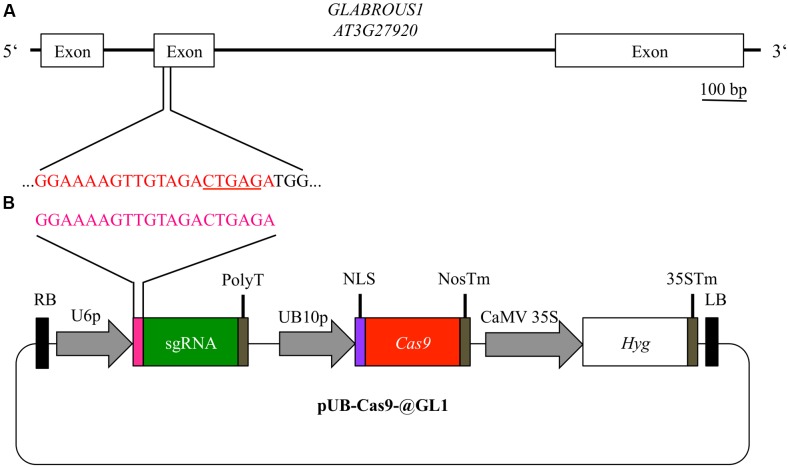
**Experimental design for disruption of the *GL1* gene**. For targeting the *GLABROUS1* gene with the Cas9 system, we selected a 20 bp target site (red) in the second exon of the *GL1* gene **(A)**. The target site contains a DdeI restriction site (underlined) spanning the area where Cas9 cleaves the dsDNA. In order to induce mutations, *Arabidopsis* WT plants were transformed with a single T-DNA construct pUB-Cas9-@GL1 **(B)** containing the sgRNA (green) with the target motive (pink) under the control of the *U6-26* promoter (U6p), the *Cas9* gene (red) controlled by the *UBIQUITIN10* promoter (UB10p) and the selection marker hygromycin (Hyg). Gene sizes in the vector map are not to scale. RB, right T-DNA border; PolyT, poly thymidine transcription terminator; NLS, nuclear localization signal; NosTm, nopaline synthase terminator; CaMV 35S, *Cauliflower Mosaic Virus* 35S promoter; 35STm, 35S terminator; LB, left T-DNA border.

Using the vector system described above, we generated the binary T-DNA vector pUB-Cas9-@GL1 (**Figure 2B**) to introduce a targeted gene knockout in the *GL1* gene of *Arabidopsis* by the Cas9 system. pUB-Cas9-@GL1 contained all the features of pUB-Cas9 and additionally the sgRNA cassette with the *U6-26* promoter, a 20 bp protospacer sequence targeted against *GL1* and the sgRNA scaffold. We selected a targeting site that is located at the beginning of the second exon of the gene (5′-GGAAAAGTTGTAGACTGAGA-3′; **Figure 2A**).

### Chimeric Trichome Patterns Appear in the T2 Generation

**FIGURE 3:**
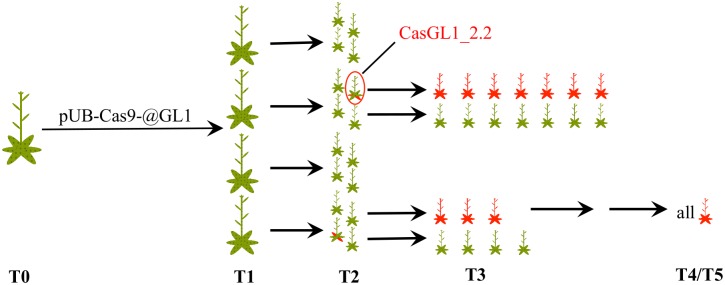
**Schematic overview of analyzed plants**. We analyzed several independent lines transformed with pUB-Cas9-@GL1 over five generations. This scheme shows the number of analyzed plants up to the T3 generation and the appearance of chimeric and glabrous plants over time (red areas = glabrous areas). The analyzed T4 and T5 plants were all glabrous. The plant CasGL1_2.2 (red circle) is the chimeric plant that was analyzed with a restriction fragment length polymorphism assay (see **Figure 4**).

We visually analyzed trichome appearance over five generations in different independent transformant lines (**Figure 3**). In the following, all examined plants will be named CasGL1 followed by an unique number containing information about their progenitor and referring to the scheme in **Figure 3**. As an example, the T2 plant marked by the red circle in **Figure 3** is named CasGL1_2.2 because it derives from the T1 plant number 2 (first number; CasGL1_2) and is the second out of four analyzed plants from that progenitor in that generation. The seven plants in its progeny are then called CasGL1_2.2.1-7.

**FIGURE 4:**
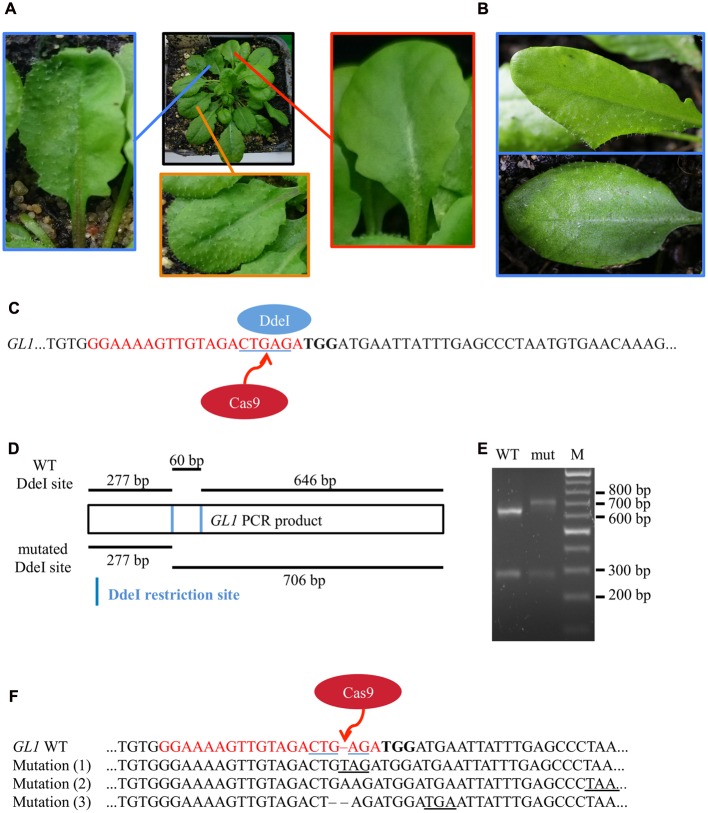
**Mutation analysis in chimeric T2 generation plants**. **(A)** Several T2 plants displayed a combination of partly glabrous (blue), to completely glabrous (red), or WT-like leaves (orange). The trichome pattern on the partly glabrous leaves was different on individual chimeric leaves in different plants **(B)**. The restriction fragment length polymorphism assay was used to verify mutations on gDNA level. **(C)** As mutations induced by Cas9 nuclease disrupt a DdeI restriction site (blue underlined) in the *GL1* target site (red), digestion of *GL1* PCR amplicons of mutant and WT alleles yield a different restriction pattern. **(D)** We amplified the *GL1* gene from genomic DNA from the glabrous leaf shown in **(A)** (mut) and a WT leaf and digested it with DdeI. The resulting DNA fragments **(E)** match the length expected for WT (277 and 646 bp) and mutant (277 and 706 bp) alleles, the 60 bp band in the WT allele is not retained by the gel (M, Marker). Sequencing of the *GL1* gene revealed three different InDel mutations in the glabrous leaf **(F)** 3 bp upstream of the PAM (bold) within the sgRNA target sequence (red), all causing frameshifts and the formation of premature STOP codons (underlined).

After transformation of WT plants with the construct pUB-Cas9-@GL1, we visually analyzed four T1 generation plants but could not detect any completely or partly glabrous plants. Four descendants of each T1 plant were analyzed in the T2 generation. While most plants still displayed WT-like trichome patterns, two plants from two independent T1 lines displayed a chimeric phenotype (CasGL1_2.2 and CasGL1_4.3). That is, these plants had few either completely or partly glabrous leaves, which hints at a disruption of both alleles of the *GL1* gene in the glabrous tissues (**Figures 4A,B**).

### Restriction Fragment Length Polymorphism Assay Indicate Mutations in Glabrous Leaves

To verify that the genomic DNA from glabrous leaves indeed contained InDels in the *GL1* gene in the targeted sequence, we used a restriction fragment length polymorphism assay. We made use of the DdeI restriction site within the target site of the Cas9 nuclease that was expected to be destroyed by InDel mutations (**Figure 4C**). We amplified the genomic region that spans the target site from genomic DNA of a WT leaf and a glabrous leaf of plant CasGL1_2.2, respectively, and digested the PCR product with DdeI (**Figure 4D**). The PCR product from the glabrous leaf was resistant to DdeI digestion, which provided evidence for a mutation in the DdeI target site (**Figure 4E**). The PCR product was also subcloned into pJET1.2 and seven clones were sequenced (**Figure 4F**). Three different InDels were found 3 bp upstream of the PAM motive, four clones out of seven contained an adenine insertion, two a thymine insertion and one clone had a guanine deletion. As both alleles of *GL1* have to be disrupted to generate a glabrous phenotype, the high proportion of clones with the adenine insertion suggests that this mutation happened at early developmental stages or in the previous generation, while the thymine insertion and guanine deletion happened later in independent progenitor cells on the other allele disrupting trichome formation in that specific leaf.

### Completely Glabrous Plants Emerge in the T3 Generation

**FIGURE 5:**
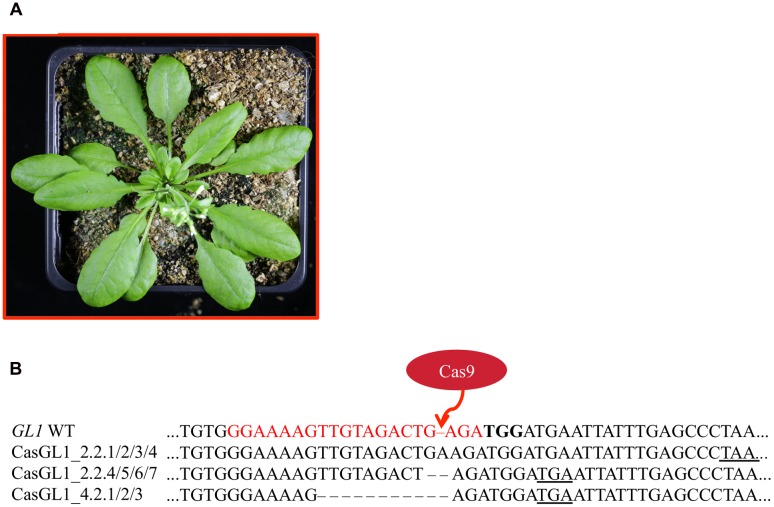
**Glabrous plants can be generated within three generations**. Completely glabrous plants **(A)** were obtained in the T3 generation. Plant CasGL1_4.2.1 is shown as a representative example. Sequencing of the glabrous plants revealed three different frameshift mutations **(B)**, which all lead to premature stop codons (underlined). The PAM is marked in bold letters, the sgRNA target site in red letters.

Four T2 plants (chimeric plant CasGL1_2.2, three WT trichome pattern plants CasGL1_2.4, CasGL1_4.2 and CasGL1_4.4) were carried to the next generation. While two T3 lines displayed normal trichome patterns, all descendants of the chimeric line CasGL1_2.2 and of the WT-like line CasGL1_4.2 were completely glabrous (**Figures 3** and **5A**). In the glabrous lines, trichomes were neither visible on leaves nor on stems. We did not observe any difference in growth or development.

### Loss of Function Is Caused by Small InDels

To analyze the nature of the generated mutations, we amplified the *GL1* gene from the glabrous plants and sequenced the PCR products (**Figure 5B**). As control, we also sequenced the *GL1* gene from the non-glabrous T3 plants CasGL1_4.4.1 and CasGL1_2.4.1 (Supplementary Figure S4). While the control plants did not show any changes in the *GL1* sequence, the three analyzed descendants of line CasGL1_4.2 showed a 10 bp deletion. The other descendants from the chimeric T2 line CasGL1_2.2 showed either a homozygous adenine insertion (three out of seven analyzed plants, 3/7 plants), a homozygous guanine deletion (3/7 plants) or a biallelic mutation (+A/-G; 1/7 plants). All mutations generated a premature stop codon. The high amount of homozygous mutant plants in the T3 generation cannot be explained by Mendelian segregation only and might hint at allelic conversion events.

### Presence of *Cas9* Gene Does Not Influence the Stability of the Mutation

**FIGURE 6:**
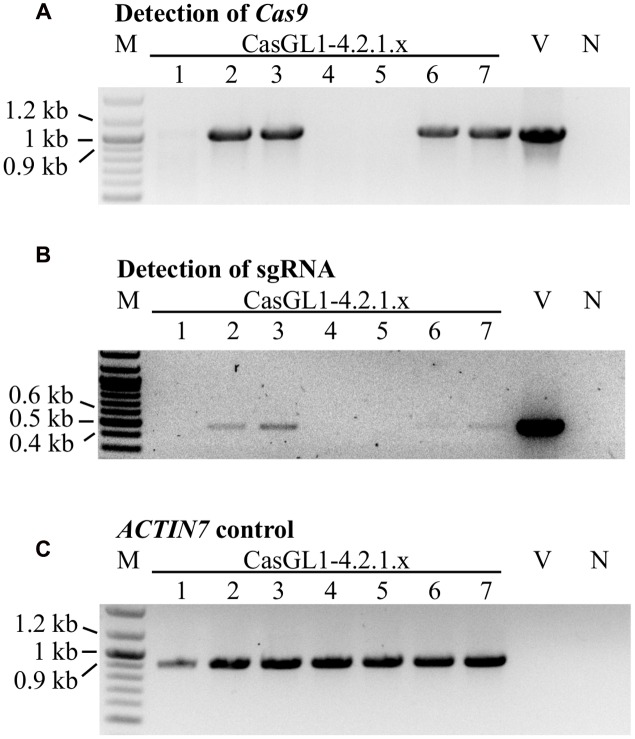
**Segregation analysis of the Cas9 T-DNA cassette in glabrous T4 generation plants**. Seven Cas9-generated glabrous plants of the T4 generation (CasGL1_4.2.1.1-7) were analyzed by PCR for the presence of the *Cas9* gene (expected size: 1 kb, **A**) and the sgRNA cassette (expected size: 0.49 kb, **B**) using the *Arabidopsis ACTIN7* gene as DNA quality control (expected size: 0.92 kb, **C**). Images were color inverted for better visibility. M, marker; V, vector control (pUB-Cas9-@GL1); N, negative control with water.

To test whether the mutations were stably inherited, we brought the glabrous plant CasGL1_4.2.1 with the 10 bp deletion in the *gl1* gene into the T4 and T5 generation and visually analyzed the plants for reappearance of trichomes. All progeny maintained their glabrous phenotype. We also investigated whether it was possible to obtain stably mutated plants that do not carry the Cas9 cassette in the genomic DNA anymore. Therefore, we grew the T4 generation plants on non-selective medium to recover both plants with the T-DNA cassette (including Cas9 and the sgRNA) and plants that lost the cassette by segregation. The genomic DNA of seven plants was analyzed by PCR for the presence of the Cas9 and the sgRNA gene (**Figure 6**). Three out of seven plants had lost the T-DNA with Cas9 and sgRNA but maintained their glabrous phenotype. This shows that the T-DNA is independently inherited from the generated mutation and the presence or absence of Cas9 does not influence a Cas9-generated mutation.

### No Off-Targets were Detected in Closest Homologous Sequence

A common issue for Cas9-based generation of mutants is the possibility of off-target cleavage of similar regions in the genome (Cho et al., 2014). We analyzed six glabrous descendants of the plant CasGL1_4.2.1 (T4 generation) for mutations in the closest homologous site to our target sequence. This site contains only one mismatch near the 5′ end of the sgRNA target sequence and lies in the gene *AT1G08810* (5′-GCAAAAGTTGTAGACTGAGA-3′, mismatch is underlined), which encodes for the putative Myb-type transcription factor MYB60. Sequencing of genomic DNA did not reveal mutations in this gene (Supplementary Figure S5).

## Discussion

### Positive Selection by Regaining Glufosinate Resistance Is Not a Suitable Marker for Cas9-Induced Mutagenesis

In this study, we compared two visual markers for monitoring the efficiency of Cas9-induced mutations. First, we aimed at repairing a non-functional *BAR* gene *in planta* by removing a premature STOP codon using a sgRNA directed against the mutated site (**Figure 1**). While we were able to show Cas9-induced mutations in that site in the T2 generation (**Figure 1C**), we could not explain the discrepancy between the high percentage of plants resistant to glufosinate (over 95%, Supplementary Figure S1) and the low occurrence of edited sequences in the genome of resistant plants (Supplementary Figure S2). A likely explanation is that few cells carrying an active allele of the *BAR* gene are sufficient for conferring systemic glufosinate resistance to the plant. It has been shown recently in cotton that small amounts of the PAT protein are sufficient to provide sufficient glufosinate resistance in regard to visual symptoms (Carbonari et al., 2016). Additionally, the PAT protein consists of only 183 amino acids (app. 20 kDa) and is therefore small enough to be transported through plasmodesmata between cells (Crawford and Zambryski, 2001) and perhaps even through the phloem (Stadler et al., 2005). This means that few cells producing functional PAT might be able to supplement many cells with functional PAT protein and thus providing systemic resistance. Consequently, we did not isolate plants with a fully repaired *BAR* gene with the same genotype in all cells. The observation that Cas9 induced mutations occurred in the plants at the somatic level, suggests that likely those genetic changes also occur in germline cells. Germline mutations would be passed to the next generation and sequencing a higher amount of plants might have therefore ultimately led to detection of completely mutated plants. However, as the stringency of glufosinate selection is low, at least in the standard concentration used in our work, a possible usage of this system to compare the efficiency of different Cas9 variants or expression vectors is not recommended, as differences in the efficiency of Cas9 repair would not be detectable.

### The Cas9 System Is a Highly Efficient Method to Generate Independent *gl1* Knockout Lines

A non-invasive alternative to visually track Cas9 nuclease activity in *Arabidopsis* is the disruption of the *GL1* gene. Therefore, we designed a sgRNA targeting the second exon of the gene. This site was favored for several reasons. First, no completely redundant sequence could be found in the genome of *Arabidopsis*. Second, a frameshift mutation due to small insertion or deletions (InDels) caused by the error-prone NHEJ was likely to have more drastic effects at the beginning of the gene. Third, the target site is located in one of the two MYB-type DNA-binding domains and mutations in these domains lead to more drastic phenotypes compared to mutations elsewhere in the gene (Hauser et al., 2001). Fourth, a DdeI restriction enzyme site overlapped with the cutting site. Thus, we decided to detect mutations by screening for the loss of the restriction enzyme recognition site.

In this report, we could show that the Cas9 system was highly efficient for generating knockouts in the *GL1* gene. Using a single sgRNA and a *Chlamydomonas reinhardtii* codon optimized *Cas9* gene (**Figure 2B**), we found that 50% of our analyzed T1 lines contained glabrous progenitors (**Figure 3**). In total, we were able to generate four different homozygous/biallelic glabrous lines. This was achieved in the T3 generation by screening a very limited amount of plants. Screening a higher amount of plants would have probably yielded knockouts even in the earlier generation, as shown by Cas9 experiments of other groups (Fauser et al., 2014; Feng et al., 2014). This puts the application of Cas9 technology within the reach of every laboratory equipped with basic molecular biology equipment.

The high frequency of homozygous mutants might be caused by allelic gene conversion (Li et al., 2014; Yoshimi et al., 2014), meaning that one allele is mutated first and then serves as repair template for the DSB in the second allele. Therefore, producing homozygous mutants with Cas9 can be more efficient than expected by chance.

All observed mutations were small InDels that caused a shift in the reading frame and thus the generation of premature STOP codons (**Figures 4F** and **5B**). These small InDels are typical for error-prone repair of the induced DSB by the NHEJ pathway (Salomon and Puchta, 1998; Puchta and Fauser, 2014).

Moreover, targeting the *GL1* gene allowed us to visualize both homozygous plants and chimeric leaves (**Figure 4A**). We suggest that the partially glabrous phenotype of the chimeric leaves could be used in order to track cell lineages during leaf development, which might ultimately lead to a model of cell migration through space and time (Kalve et al., 2014). Another advantage of chimeric mutations is the possibility to study the function of essential genes *in vivo* by direct comparison of mutated and non-mutated parts within the same plant and thus overcome the problem of lethality of complete knock-out plants. However, it has to be taken into account that the CRISPR/Cas9 system is constitutively active in plants under the control of the *UBIQUITIN10* promoter and new mutations can thus occur in every cell at any time. A precise delivery of Cas9 only to certain parts of a plant (e.g., by transient *Agrobacterium* leaf infiltration or tissue-specific, inducible promoters) might therefore be the favorable approach for such intraplant-comparisons.

### Transgene-Free Plants Can Be Generated with the Cas9 System

A major issue for the acceptance of genetically modified crops is the introduction of transgenes into crop plants by random integration of a T-DNA (Ishii and Araki, 2016). The Cas9 system is also classically introduced as T-DNA cassette. However, since allelic point mutations are established on different genomic loci, the T-DNA containing the Cas9 components can be removed by backcrossing. We could show via PCR analysis that some of the glabrous plants in the T4 generation had actually lost the T-DNA cassette over time while still keeping the mutation in the *GL1* gene (**Figure 6**). This is in accordance with results from other groups that generated transgene-free mutated rice (Xu et al., 2015) or *Arabidopsis* (Feng et al., 2014) plants. This underlines the opportunities for the introduction of new traits in transgene-free crop plants that are given by the Cas9 system and other targeted nucleases, such as TALENs (Wang et al., 2014). As long as these new traits are only created by allelic point mutations and not by integration of foreign transgenes that cannot be introgressed by genetic crosses, site-directed mutations are not distinguishable from mutations generated by natural processes or breeding and are hence likely to be not subject to regulatory assessments in many countries (Wolt et al., 2016). If applicable, transient delivery systems for Cas9 RNA and corresponding sgRNA (Zhang et al., 2016), or preassembled protein–RNA complexes (Woo et al., 2015) facilitate the genomic editing, without DNA integration into the target genome at any time.

### Off-Targeting Effects were Not Detectable in Plants

One potential pitfall of the Cas9 system is the possibility for off-target mutations to occur in sequences highly similar to the target site (Fu et al., 2013). In plant research, off-target mutations are highly problematic if the generated mutants are used to characterize gene functions. We targeted the DNA binding helix 3 of the MYB transcription factor GL1. Since this domain is highly conserved on the DNA sequence level in the R2R3-MYB gene family (Jia et al., 2004), off-targeting was possible. We used the CasOFF-Finder program (Bae et al., 2014) to detect putative off-target sites. While in *Arabidopsis* there is no other DNA sequence perfectly matching to the target site, three gene sequences have only one mismatch to the sgRNA target site against *GL1* used in this study. Two of them have a mismatch in the 3′-end of the protospacer motive. This region is called seed sequence. A mismatch in the seed sequence strongly reduces the affinity of the Cas9-sgRNA complex toward its off-target (Semenova et al., 2011; Jinek et al., 2012). Therefore, we focused on the third putative off-target site, where the mismatch is near the 5′-end of the protospacer. However, sequencing of this gene in six glabrous T4 generation plants did not show off-target mutations (Supplementary Figure S5). This result is in line with previous observations that off-targeting does not occur at high rates in *Arabidopsis* as shown by in-depth whole genome analysis of *Arabidopsis* Cas9-edited lines where no off-targets in highly homologous sequences could be detected (Feng et al., 2014). Additionally, it has to be taken into account that off-target mutations can be removed in plants by backcrossing to the parental line.

### *GL1* Is a Suitable Marker for Visual Detection of Mutation Efficiency

Even though successful gene knockout using the Cas9 system has already been reported for *Arabidopsis* and other plant species(Bortesi and Fischer, 2015), our study provides a direct readout of the mutagenic process in living plants throughout the plant development by directing Cas9 to *GL1*. Previous visual markers mostly relied on fluorescence proteins (Jiang et al., 2014) or on the GUS reporter system (Mao et al., 2013), which had to be transformed and expressed in the plant. Instead, the disruption of trichome formation allows following the progress of mutagenesis without having pleiotropic effects on the development of the plant (Oppenheimer et al., 1991). The glabrous lines generated in this work now provide the opportunity to detect heterozygous mutations using a reverse approach by repairing the *GL1* gene via Cas9. Especially the lines CasGL1_4.2.1/2/3 with the 10 bp deletion is of interest for HR based repair approaches. We propose that trichome loss can be exploited in all trichome-containing crop species as a marker for mutagenesis efficiency if the genes for trichome initiation are described [e.g., the *Glabrous Rice 1* gene in rice (Li et al., 2012) or the *Wooly* gene in tomato (Yang et al., 2011)].

## Conclusion

In this study, we confirmed and showed in detail that the Cas9 system can be used to introduce small mutations with precision in the genome of *Arabidopsis.* The mutations are stably maintained in the genome even when the *Cas9* gene is lost. The efficiency of obtaining plants carrying homozygous mutation was higher than expected by mutation frequency alone. Thus, Cas9 mediated genome editing is a powerful and easy to use system for genome editing in *Arabidopsis.* GL1 is a reliable marker for observing mutation efficiency in living plants. Our marker approach might be of use for comparing different mutagenesis approaches, to enhance the mutagenesis efficiency, and finally for optimization of the Cas9 toolbox.

## Author Contributions

FH, OM, ME, and AW designed the study. FH carried out all laboratory experiments except the design of the codon-optimized Cas9 and cloning of the vectors CasYFP and pKS Chlamy dual (done by AG and PH). FH, OM, ME, and AW interpreted the data. FH, OM, ME, and AW wrote the manuscript with contributions of AG and PH.

## Conflict of Interest Statement

The authors declare that the research was conducted in the absence of any commercial or financial relationships that could be construed as a potential conflict of interest.
