# Supplementary material for: An Efficient Visual Screen for CRISPR/Cas9 Activity in Arabidopsis thaliana
Source: Front Plant Sci. 2017 Jan 24;8:39. doi: 10.3389/fpls.2017.00039 (PMC5258748; doi:10.3389/fpls.2017.00039)
Supplement: Supplementary file 1 [file Data_Sheet_1.docx]

Supplementary Material

**An efficient visual screen for CRISPR/Cas9 activity in *Arabidopsis thaliana***

Florian Hahn, Otho Mantegazza, André Greiner, Peter Hegemann, Marion Eisenhut, Andreas P.M. Weber*

*** Correspondence:** Corresponding Author: [Andreas.Weber@uni-duesseldorf.de](mailto:Andreas.Weber@uni-duesseldorf.de)

# Supplementary Figures and Tables

## Supplementary Figures


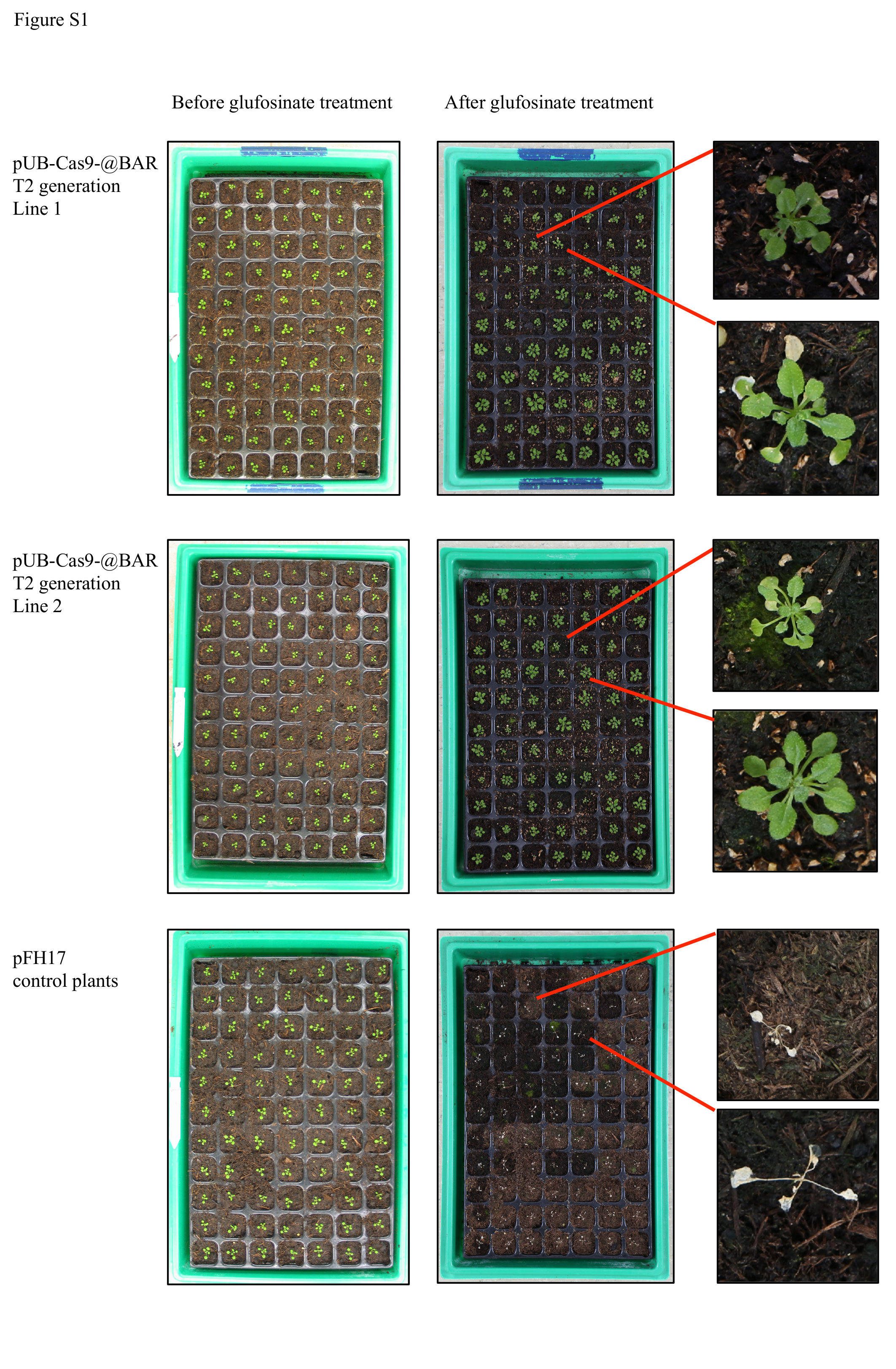


**Fig. S1: Glufosinate treatment of T2 generation plants transformed with the vector pUB-Cas9-@BAR**. We grew two independent lines of *Arabidopsis* T2 transformants with the vector pUB-Cas9-@BAR on soil. Repetitive glufosinate spraying after three weeks of growth yielded in over 95% surviving plants in both transformant lines with green glufosinate-resistant tissue. Still, some plants showed partly yellowish leaf tissue in variable amounts (compare detail pictures on the right). In contrast, the background line carrying the null *bar*-1 allele in homozygosis and no *Cas9* or sgRNA did not survive the glufosinate treatment (pFH17, bottom).


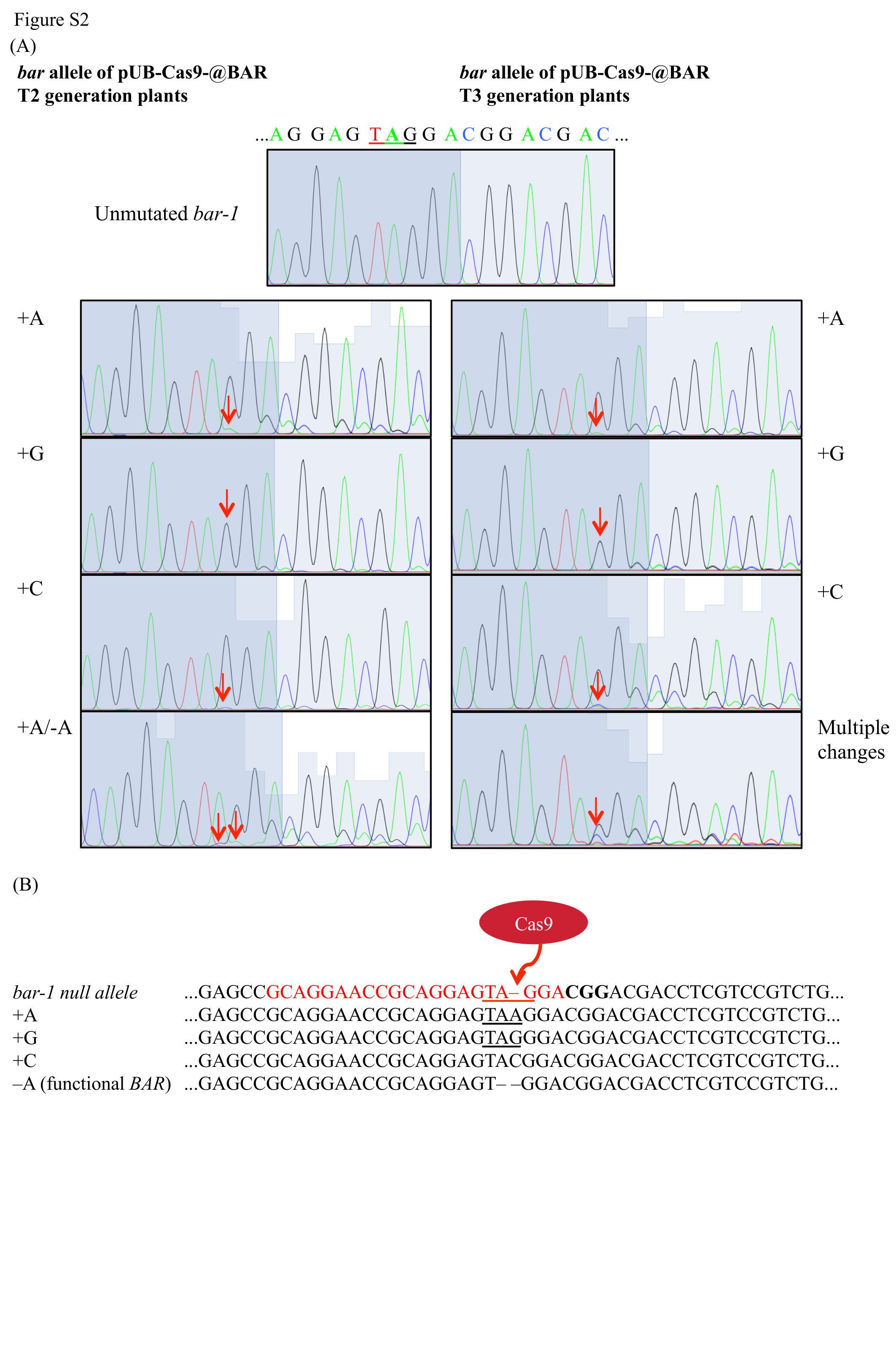


**Fig. S2: Representative sequencing results of glufosinate resistant plants**. We sequenced the *bar* gene in plants of the T2 and the T3 generation (A) that survived the glufosinate treatment. Next to plants showing an adenine deletion, which restores the reading frame (compare Figure 1C), most of the plants showed either no change (top), 1 bp insertions, multiple changes or no change, all of them not restoring a functional reading frame (B) but still resulting in premature STOP codons (underlined). A = adenine, C = cytosine, G = guanine.


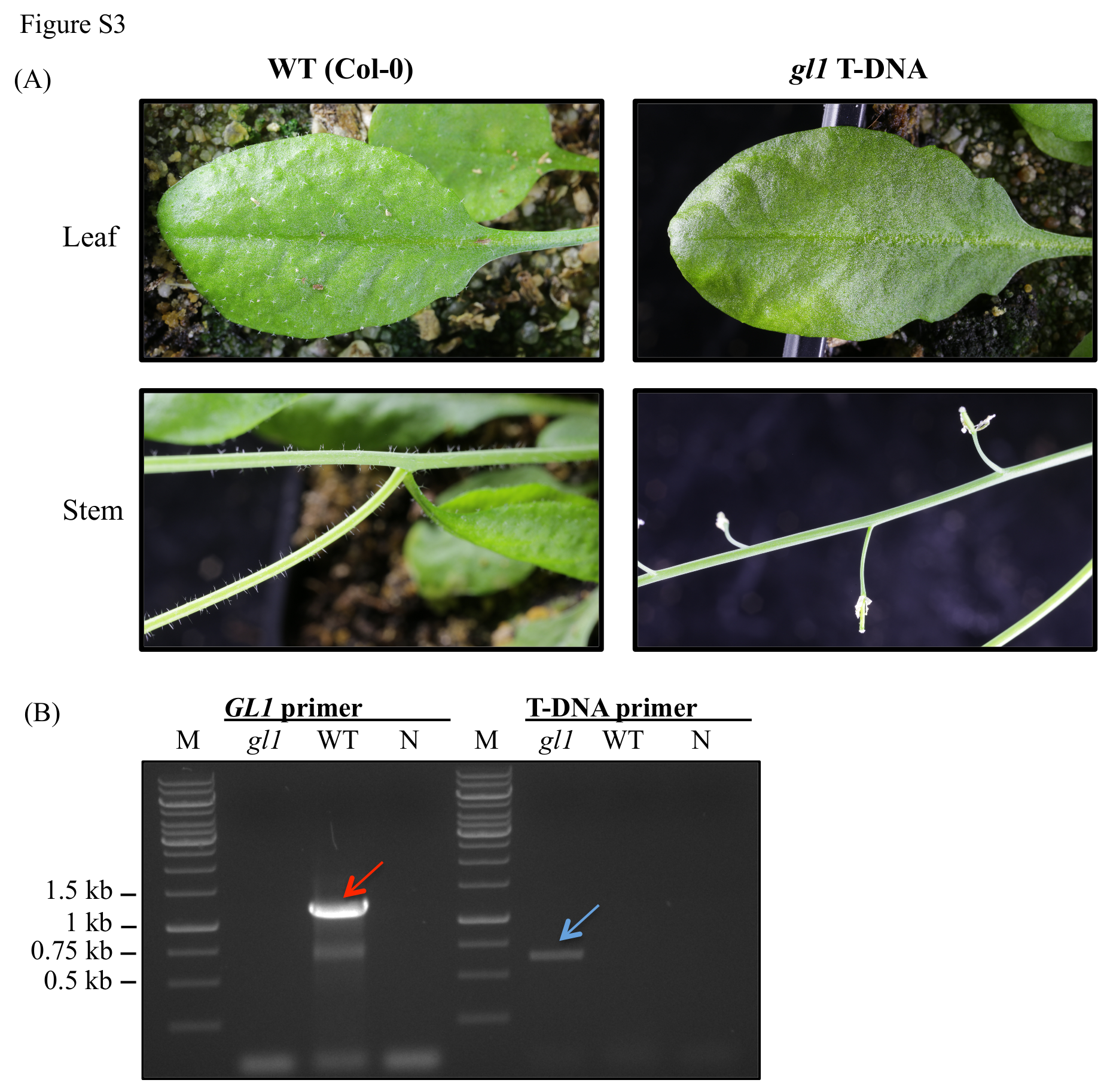


**Figure S3: Visual phenotype and homozygosity proof of *gl1* T-DNA mutant plants. (A)** We compared trichome formation of an *Arabidopsis* WT Col-0 plant and a *gl1* T-DNA knockout line. While WT plants (left) show dense trichome growth on leaves and stems, the *gl1* plants (right) display a glabrous phenotype. (B) Homozygous T-DNA insertion in the knockout line (*gl1*) was additionally confirmed with PCR using gene specific primers (left, expected product size 1267 bp) and T-DNA specific primers (right, expected product size 589-889 bp) on leaf gDNA. WT Col-0 gDNA (WT) was used as comparison; no DNA was used as negative control (N). The gene specific PCR product can only be found in the WT sample (red arrow), the T-DNA product only in the T-DNA gDNA (blue arrow). M = Marker


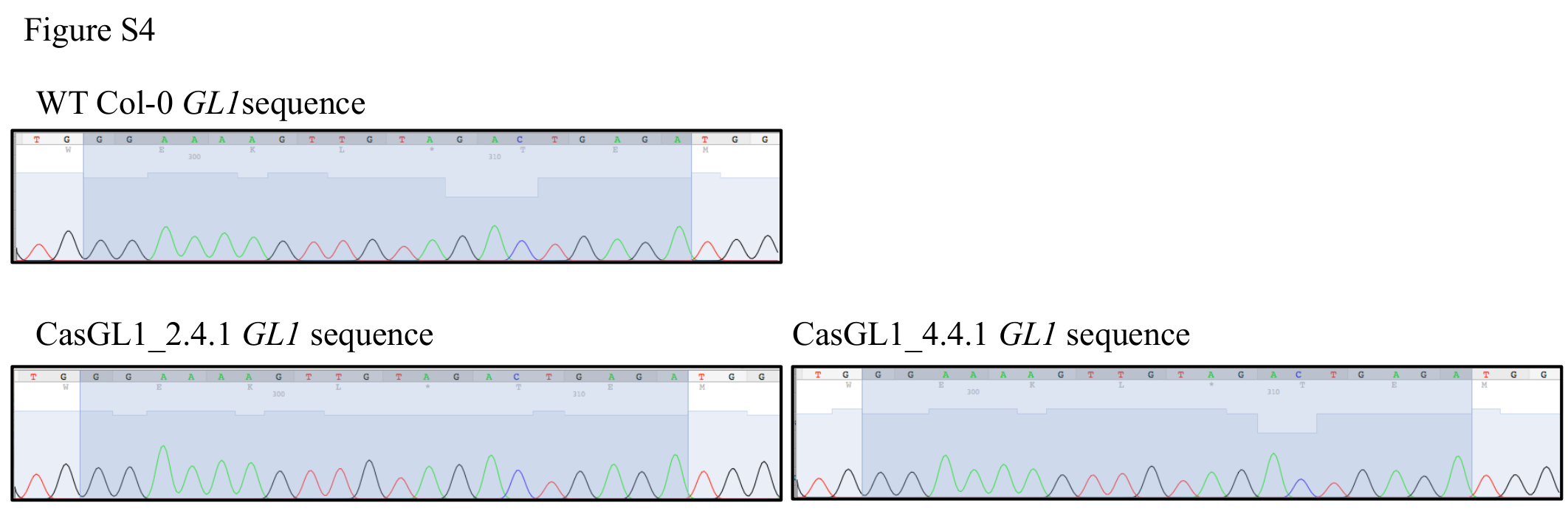
 **Fig. S4: *GL1* sequence analysis of a non-glabrous T3 plants**. Two T3 plants (CasGL1_2.4.1 and CasGL1_4.4.1) transformed with the vector pUB-Cas9-@GL1 were analyzed for mutations in the *GL1* gene. Therefore, the gene was amplified via PCR and sequenced. WT *Arabidopsis* Col-0 DNA was used as a control. No mutations could be detected on sequence level.


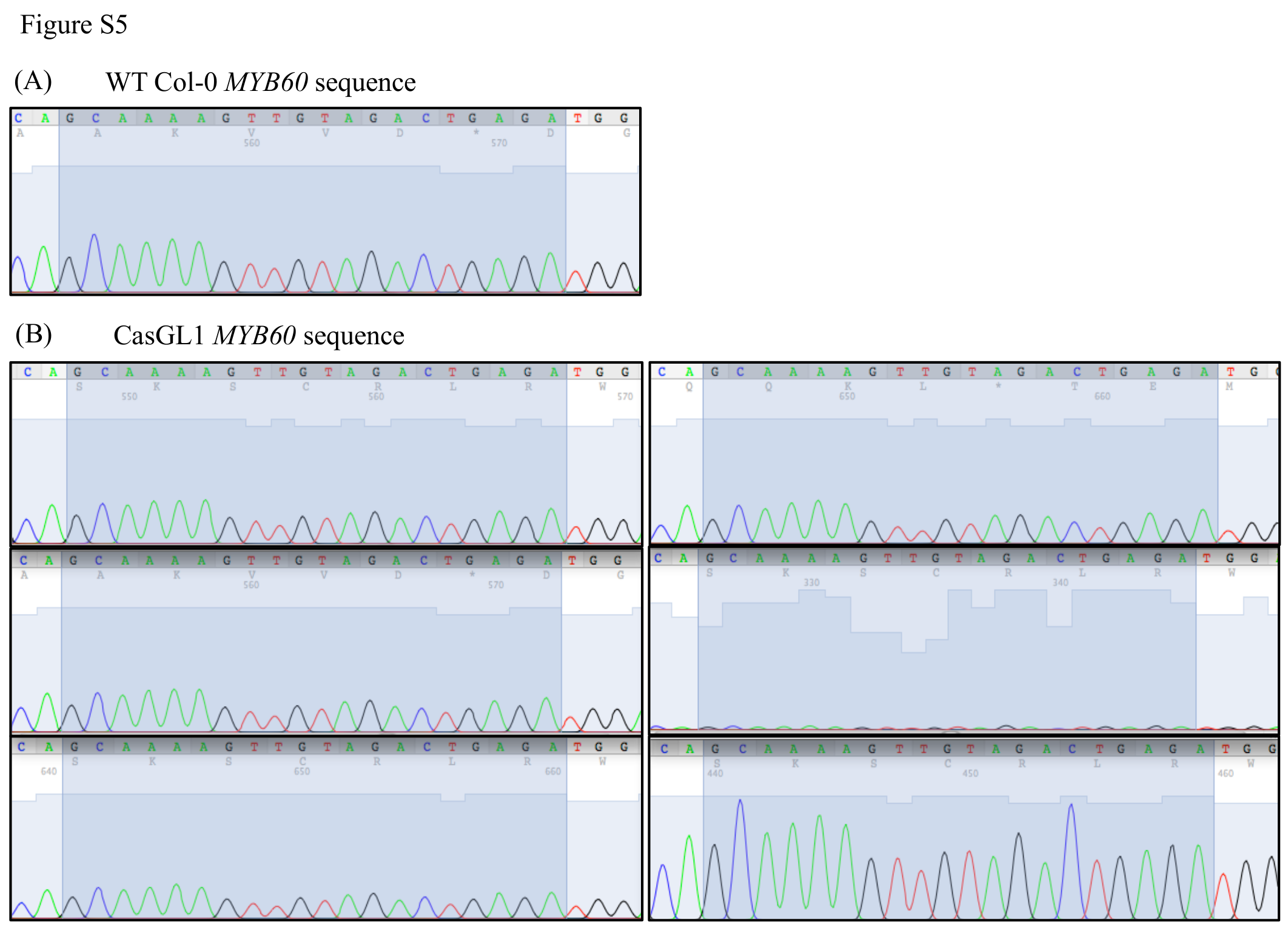


**Fig. S5: Sequence analysis of a putative pUB-Cas9-@GL1 off-target site in T4 glabrous plants**. Six Cas9-generated glabrous plants of the T4 generation (CasGL1_4.2.1.1/4/5 and CasGL1_4.2.2.1/3/4) were analyzed for mutations in a highly homologous off-target sequence in the gene *AT1G08810* (*MYB60*). Therefore, the gene was amplified via PCR and sequenced. WT *Arabidopsis* Col-0 DNA was used as a control (A). No mutations could be detected on the putative off target site in the six glabrous plants (B).

## Supplementary Tables

**Table S1: Primer sequences**

| Primer name | Sequence (5‘🡪3‘) |
| --- | --- |
| FH5 | GGGGACAAGTTTGTACAAAAAAGCAGGCTTCACCTATCGATCATGAGCGGAGAAT |
| FH6 | GGGGACCACTTTGTACAAGAAAGCTGGGTGATCAGCTTGCATGCCGGT |
| FH14 | caccAAGCTTCGTTGAACAACGGAAA |
| FH15 | caccGCTAGCGAAGGGACAATCACTACTTC |
| FH16 | caccgctagcgggtcttcgagaagacctgttt |
| FH18 | caccactagtttaaaaaaatttgcaccgact |
| FH21 | cgggaattcgattcaccgAAGCTTCGTTGAACAACG |
| FH22 | cgaagacccCGAAGGGACAATCACTACTTC |
| FH23 | gtcccttcgGGGTCTTCGAGAAGACCTG |
| FH24 | caggcggccgcgaattcaAGATCTTCCGGATGGCTC |
| FH25 | CACCTATCGATCATGAGCGGAGAA |
| FH26 | cggacgaggtcgtccgtccTACTCCTGCGGTTCCTGC |
| FH27 | gccgcaggaaccgcaggag**tAg**gacggacgacctcgtc |
| FH28 | CACCATCAGCTTGCATGCCGG |
| FH35 | TTCGGGAAAAGTTGTAGACTGAGA |
| FH36 | AAACTCTCAGTCTACAACTTTTCC |
| FH39 | ttaacagctcgagtgcggTATCGATCATGAGCGGAGAATTAAG |
| FH40 | acgaacgaaagctctgcaATCAGCTTGCATGCCGGT |
| FH41 | aaacgacggccagtgccaGAATTGGGCCCGACGTCG |
| FH42 | tactgactcgtcgggtacCAAGCTATGCATCCAACGCG |
| FH61 | CTGCCCAACGAGAAGGTGC |
| FH145 | TTCGGcaggaaccgcaggagtAgga |
| FH146 | AAACtccTactcctgcggttcctgC |
| FH154 | ATGAGCCCAGAACGACGC |
| FH155 | AGATTTCGGTGACGGGCA |
| FH158 | ATGAAAAAGCCTGAACTCACC |
| FH159 | GGTTTCCACTATCGGCGAG |
| FH189 | CTCTCACACACACACACAGACA |
| FH190 | GTTTGCACGACTAATACACTTATG |
| FH201 | agtcggacaggcggttgatg |
| FH214 | AAAGACCATCTGAATTTGGGG |
| FH215 | TAAGCACGTGTCACGAAAACC |
| FH258 | GATCAGTTCCCACCAACACT |
| FH259 | TGTCGAAGAAGTTTGCAGC |
| NH117 | GGGGACAAGTTTGTACAAAAAAGCAGGCTACCATATGGCCAGCAGCCCCCC |
| NH119 | GGGGACCACTTTGTACAAGAAAGCTGGGTAttaGTCGCCGCCCAGCTGCGA |
| P16 | CACAGTTCGATAGCGAAAACC |
| P49 | tagcatctgaatttcataaccaatctcgatacac |
| P67 | TTCAATGTCCCTGCCATGTA |
| P68 | TGAACAATCGATGGACCTGA |
| P98 | CGATTTTCTGGGTTTGATCG |
